# Temporal Dynamics of Chronic Inflammation on the Cecal Microbiota in IL-10^-/-^ Mice

**DOI:** 10.3389/fimmu.2020.585431

**Published:** 2021-02-16

**Authors:** Anne-Marie C. Overstreet, Amanda E. Ramer-Tait, Jan S. Suchodolski, Jesse M. Hostetter, Chong Wang, Albert E. Jergens, Gregory J. Phillips, Michael J. Wannemuehler

**Affiliations:** ^1^ Department of Veterinary Microbiology and Preventive Medicine, Iowa State University, Ames, IA, United States; ^2^ Department of Food Science and Technology, University of Nebraska, Lincoln, NE, United States; ^3^ GI Laboratory, Department of Small Animal Clinical Sciences, Texas A&M University, College Station, TX, United States; ^4^ Department of Pathology, University of Georgia, Athens, GA, United States; ^5^ Veterinary Diagnostics and Production Animal Medicine, Iowa State University, Ames, IA, United States; ^6^ Veterinary Clinical Science, Iowa State University, Ames, IA, United States

**Keywords:** IBD, IL-10, enterobacteriaceae, colitis, microbiome, temporal dynamic changes, dysbiosis, cytokine response

## Abstract

The intestinal microbiota is a critical component of mucosal health as evidenced by the fact that alterations in the taxonomic composition of the gastrointestinal microbiota are associated with inflammatory bowel diseases. To better understand how the progression of inflammation impacts the composition of the gastrointestinal microbiota, we used culture independent taxonomic profiling to identify temporal changes in the cecal microbiota of C3Bir IL-10^-/-^ mice concomitantly with the onset and progression of colitis. This analysis revealed that IL-10^-/-^ mice displayed a biphasic progression in disease severity, as evidenced by histopathological scores and cytokine production. Beginning at 4 weeks of age, pro-inflammatory cytokines including TNF-α, IFN-γ, IL-6, G-CSF, and IL-1α as well as chemokines including RANTES and MIP-1α were elevated in the serum of IL-10^-/-^ mice. By 19 weeks of age, the mice developed clinical signs of disease as evidenced by weight loss, which was accompanied by a significant increase in serum levels of KC and IL-17. While the overall diversity of the microbiota of both wild type and IL-10^-/-^ were similar in young mice, the latter failed to increase in complexity as the mice matured and experienced changes in abundance of specific bacterial taxa that are associated with inflammatory bowel disease in humans. Collectively, these results reveal that there is a critical time in young mice between four to six weeks of age when inflammation and the associated immune responses adversely affect maturation of the microbiota.

## Introduction

Inflammatory bowel diseases (IBD), which include Crohn’s disease (CD) and ulcerative colitis (UC), are chronic immunologically-mediated disorders affecting the gastrointestinal (GI) tract. The current hypothesis for the pathogenesis of IBD involves a complex interplay between the mucosal immune system, environmental factors and the intestinal microbiota in a genetically susceptible host (1–4). The role that enteric bacteria play in the initiation, progression, and maintenance of the GI inflammatory process represents a particularly active area of investigation. Studies using rodent models as well as characterization of IBD patients, strongly implicate the gut microbiota as having a significant impact on the pathogenesis of colitis (5–8). For example, it has been observed that inflammatory bowel diseases correlate with dysbiosis (i.e., reduced diversity/complexity of the microbiota) (9–13). There is evidence of demonstrable changes in the abundance of specific bacterial taxa associated with IBD including an increase in Proteobacteria in humans, mice, and companion animals (14–18). In addition, a decrease in *Clostridium* cluster groups IV and XIVa have been noted in patients with active IBD (9, 13, 19, 20). These *Clostridium* cluster groups comprise a large proportion of the gut microbiota in healthy individuals and are known producers of short chain fatty acids, such as butyrate, which serve as an important energy source for colonocytes as well as regulatory molecules for regulatory T-cells (21, 22). Although it is well established that microbial-derived products activate both innate and adaptive immune cells and contribute to intestinal inflammation (23–26), a key question remains as to what extent the inflammation associated with IBD directly causes changes in the abundance of specific taxa.

To better understand the relationship between the host microbiota and immune system responses, we chose to use interleukin-10 deficient (IL-10^-/-^) mice to assess the development of GI inflammation and changes in the resident microbiota over time (i.e., 4 to 19 weeks of age) (27, 28). IL-10 is a key cytokine that facilitates immune regulation (29) and consequently IL-10^-/-^ mice develop spontaneous enterocolitis associated with the lack of regulatory T cells. The manifestation of disease can vary in severity depending on the composition of the intestinal microbiota and the genetic background of the mice (27, 30). The importance of mouse strain and the microbial composition to the development of colitis in IL-10^-/-^ mice was elegantly studied by Hart at al. In their study they transferred embryos into surrogate dams from various vendors and then examined the resulting offspring for inflammation (31). Compared to IL-10^-/-^ mice reared under conventional housing conditions, mice maintained under specific-pathogen free (SPF) conditions (i.e., absence of *Helicobacter* species) have attenuated disease while germ-free IL-10^-/-^ animals fail to develop disease (32–34). A study by Whary et al. revealed that at least two bacterial species (*Lactobacillus reuteri* and *Helicobacter hepaticus*) needed to be present in the GI microbiota before inflammation could develop (35). Mice on a C3H/HeJBir background are highly susceptible to disease compared to other IL-10^-/-^ strains such as C57BL/6J which are resistant to the development of colitis (4, 30). This is in part because the parental strain (C3H/HeJBir) is prone to spontaneous colitis due to the presence of the major colitis susceptibility locus (*Cdcs1*) on chromosome 3 (3, 36, 37).

While IL-10^-/-^ mouse models have been used to correlate differences in bacterial taxa present with the severity of inflammation (38–41), we reasoned that additional insights into the disease process can be gained from a longitudinal study that describe the dynamic changes in the microbiota over multiple time points as inflammation progresses (11, 42). To address this unknown, we used C3Bir.129P2(B6)-*Il10^tm1Cgn^*/Lt (C3Bir IL-10^-/-^) mice as a genetic model of gastrointestinal inflammation (28, 30, 43–45).

The cecal microbiota of IL-10^-/-^ mice, along with that of C3H/HeJ control animals, were characterized by 16S rRNA gene amplicon sequencing along with histological observations and measurements of serological biomarkers of inflammation to assess changes in the microbial composition and concurrent host responses between 4 and 19 weeks of age. The histological and inflammatory biomarker data indicated that mucosal inflammation in C3Bir IL-10^-/-^ mice was moderate to severe as early as 4 weeks of age. Over the course of the 15-week study, an increase in the Shannon diversity index and the number of observed bacterial species was seen only in WT mice. Specific bacterial taxa were altered in abundance in the IL-10^-/-^ mice, including a reduction in bacteria generally regarded as beneficial to the host such as members of the *Clostridium* cluster group XIVa, which is reduced in patients with IBD (13, 46). An increase in *Enterobacteriaceae* was also observed, reaching 8% of the total cecal bacterial community at 19 weeks, compared to less than 0.5% in WT mice. We also observed greater variability in the microbial composition throughout the study in the IL-10^-/-^ mice, which is consistent with recent observations in human IBD patients (11). We also used the metagenome prediction software, PICRUSt, to look at differences between the two communities (47). The results from our analysis revealed that the IL-10^-/-^ community had a greater number of predicted functions than the WT community post 4 weeks of age. These results build upon prior work evaluating the C3Bir IL-10^-/-^ mouse model and reveals a strong correlation between levels of inflammatory biomarkers (e.g., cytokines and chemokines) and the failure to develop a diverse and rich microbial community that otherwise occurs in wildtype mice (4, 38, 39).

## Materials and Methods

### Animals

Four-week-old female homozygous IL-10^-/-^ mice on a C3H/HeJBir background (C3Bir.129P2(B6)-*Il10^tm1Cgn^*/Lt) and C3H/HeJ controls were obtained from Jackson Laboratory (Bar Harbor, ME) in two separate shipments. The C3Bir IL-10^-/-^ mice were positive for both *H. hepaticus* and *Helicobacter muridarum.* As the mice from different litters were mixed with the shipping container, they were then randomly placed into cages with a maximum of five mice per cage. At Iowa State University, animals were housed under SPF conditions in Innocage**^®^** IVC cages supported by an Innovive IVC rack (San Diego CA). All mice were maintained in the same room (12:12 h dark/light cycle) and there were no other mice housed within this room. Mice were allowed *ad libitum* access to 2019S diet from Envigo (Madison, WI) and the drinking water was acidified (pH 2.8-3.2). All provisions (food, water, bedding) were autoclaved prior to use. Body weights were measured three times per week during the experiment. The mice were all routinely monitored for appearance of any clinical signs of disease (e.g., weight loss, diarrhea, rectal bleeding). All animal procedures were performed in accordance with the experimental protocol approved by the Iowa State University Institutional Animal Care and Use Committee.

### Sample Collection and Preparation

Randomly selected (i.e., from different cages) IL-10^-/-^ mice (n = 5 to 13 per time point) and the C3H/HeJ age-matched cohorts were necropsied at 4, 7, 10, 12, or 19 weeks of age. At each time point, mice were collected from a multiple of cages for each cohort to avoid assessing a cage effect. Blood was collected ***via*** cardiac puncture following euthanasia using CO_2_ asphyxiation. For DNA extraction, the cecum was aseptically excised and a portion of each cecum with contents were snap frozen in liquid nitrogen and stored at −20°C. The remaining cecal and colonic tissues from the same mice, devoid of contents, were placed in 10**%** buffered formalin for histopathologic evaluation.

### Macroscopic Assessment of Intestinal Lesions

Macroscopic lesions of the cecum were scored using the following parameters: 1) atrophy, 2) enlarged cecal tonsil, 3) diarrheic luminal contents, 4) presence of fresh blood, 5) gross thickening (edema) of tissue. Lesion severity scores were based on the number of gross lesions with a maximum score of 5, indicative of severe disease. Macroscopic lesions of the colon were similarly scored using the following parameters: 1) presence of fresh blood, 2) gross thickening (edema) of tissue, 3) diarrheic luminal contents. Lesion severity scores were based on the number of gross lesions with a maximum score of 3, indicative of severe disease.

### Histopathological Assessment

Fixed tissues were embedded in paraffin, sectioned and stained with hematoxylin and eosin (H&E) for light microscope evaluation. Stained tissue sections of cecum and colon were scored in a blinded fashion by a board-certified veterinary pathologist (JMH), as previously described (48, 49). The final histological score represented the numerical sum of all five parameters with each parameter scored from 0–5 (0= no lesion, 5= maximum severity) with a maximum cumulative score of 25. Histopathologic parameters evaluated included: 1) mucosal ulceration, 2) magnitude of lamina propria infiltration and character of inflammatory cells, 3) mucosal edema, 4) stromal collapse, 5) crypt hyperplasia.

### Serum Cytokine/Chemokine Quantification

At necropsy, blood was collected by cardiac puncture and allowed to clot for 24 h at 4°C. Serum was harvested following centrifugation of the blood for 10 min at 10,000 x *g* and was then stored at −20°C until analyzed. Measurements of cytokine and chemokine concentrations were performed using a murine multiplexed antibody array according to the manufacturer’s instructions (Millipore, Billerica, MA). The mean fluorescent intensity (MFI) values were converted to analyte concentrations using known standards and commercial software (xPONET^®^).

### Serum Amyloid A (SAA) Quantification

The concentration of the acute phase protein, serum amyloid A (SAA), in the serum was measured using a commercial ELISA kit (Tridelta Development, Ireland) according to the manufacturer’s instructions. After completion of the reaction, the absorbance was measured at 450 nm (Spectra Max 190, Molecular Devices). The concentration of SAA in the serum was calculated based on known concentrations of SAA standards, which were evaluated on the same plate as the samples. All reactions were performed in duplicate.

### DNA Extraction From Tissues and Contents

Total genomic DNA was extracted from cecal tissues and their contents using a bead beating and column extraction method (50). Samples were subjected to homogenization using zirconia beads (1:3 ratio of 0.5 mm and 0.1 mm beads) with subsequent heating to 70°C for 15 min. This step was repeated twice. The DNA was purified using a QIAamp column (Qiagen, Valencia CA) and concentrations quantified using a Nanodrop spectrophotometer (Thermo Scientific, Wilmington DE).

### 16S rRNA Gene Amplicon Sequencing

Amplicon sequencing was performed by the Research and Testing Laboratory (Lubbock, TX) using 454 pyrosequencing technology as previously described with primers forward 28F: GAGTTTGATCNTGGCTCAG and reverse 519R: GTNTTACNGCGGCKGCTG (51). Raw sequence data were screened, trimmed, filtered, denoised, and chimera depleted with default settings using the QIIME pipeline version 1.5.0 (52) and UCHIME. FASTA formatted sequences were then clustered into operational taxonomic unit (OTU) clusters with 96.5% identity (3.5% divergence) using USEARCH (53). For each cluster, the seed sequence was placed in a FASTA formatted sequence file. This file was then queried against a database of high-quality sequences derived from NCBI using a distributed.NET algorithm that utilizes BLASTN+. The BLASTn+ outputs were compiled using a.NET and C# analysis pipeline. The data reduction analysis was performed as previously described (51). Data are deposited in GenBank’s short read with accession number SRP033709.

To account for unequal sequencing depth across samples, subsequent analysis was performed on a randomly selected subset of 1,900 sequences per sample. This number was chosen to avoid exclusion of samples with lower number of sequence reads from further analysis. Alpha diversity (i.e., rarefaction) and beta diversity measures were calculated and plotted using QIIME. Differences in microbial communities between animal groups and time points were investigated using the phylogeny-based unweighted Unifrac distance metric. To determine if any groups of samples contained significantly different bacterial communities, the analysis of similarities (ANOSIM) function in the statistical software package PRIMER 6 (PRIMER-E Ltd., Lutton, UK) was used on the unweighted UniFrac distance matrix (17).

### *PICRUSt* Analysis

The software PICRUSt (Phylogenetic Investigation of Communities by Reconstruction of Unobserved States) (47) available through the Galaxy workflow framework (54, 55), was used to make functional gene content predictions based on 16S rRNA gene data present in the Greengenes database (47). Linear discriminant analysis effect size (LEfSe) (56) was used to determine difference in predicted genes and their specific KEGG orthologs at the various time-points between WT and IL-10^-/-^ mice.

### Statistical Analysis

All values were expressed as mean ± SEM. Serum cytokine/chemokine data and SAA data were normalized by log transformation. A repeated measures analysis of variance (ANOVA) model was used for the previously mentioned data, with treatment group, time and their interaction as fixed effects. The 454 pyrosequencing data were analyzed using a generalized linear mixed model, with the same fixed effects structure. T-tests were used to assess the differences between WT and IL-10^-/-^ at each time point. To test each treatment temporally, an effect comparison of time by group was used. Tukey’s t-test was used for multiple pairwise comparisons among time points. Non-parametric data were evaluated using a Wilcoxon Rank Sums test. PICRUSt was used to predict the functional capabilities of bacteria based on the 16S rRNA gene data set. LEfSe was utilized to evaluate differentially abundant bacterial taxa and predicted function between the animal groups. P-values ≤ 0.05 were considered significant for all tests. SAS software (SAS Institute Inc., Cary NC, USA) was used for all statistical calculations.

## Results

### Taxonomic Profiling Showed Altered Taxonomic Diversity Microbial Diversity in IL-10^-/-^ Mice Compared to WT Mice

**Figure 1:**
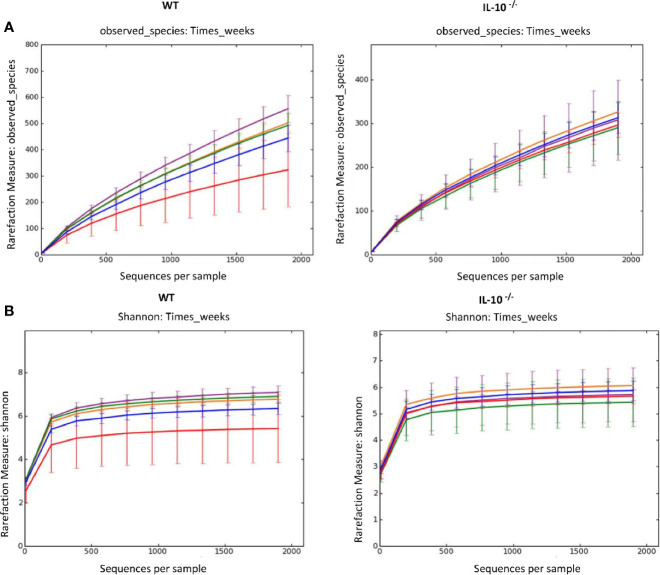
Alpha diversity measurements of cecal microbial composition. **(A)** Rarefaction analysis index and **(B)** Shannon diversity index of 16S rRNA gene sequences obtained from cecal tissue and contents. Each line represents the average of each group from IL-10^-/-^ and WT mice, as indicated. Data points are as follows: red, week 4; blue, week 7; orange, week 10; green, week 12; and purple, week 19, and error bars represent the standard deviations. The number of mice used for each strain at each time point varied as follows: at 4 weeks of age, n = 5; at 7 weeks of age, n = 10; at 10 weeks of age, n = 9; at 12 weeks of age, n = 13; at 19 weeks of age, n = 5.

To compare the composition of the GI microbiota between C3Bir IL-10^-/-^ and WT C3H/HeJ mice, 16S rRNA gene amplicon sequencing was used to identify the predominant bacterial taxa from cecal contents collected temporally from separate mice between 4 and 19 weeks of age. As the mice aged over the 19 weeks, rarefaction analysis of the cecal microbiota revealed that the number of OTUs increased in the WT mice (**Figure 1A**), as well as did the Shannon diversity index (**Figure 1B**). In contrast, the beta-diversity of the microbiota did not change in the IL-10^-/-^ mice over the 19 weeks of the study.

Comparison of the taxonomic groups between IL-10^-/-^ and WT mice revealed differences in abundance of specific phyla. Members of the Firmicutes were the most abundant in both groups of mice, although the proportion was consistently higher in WT C3H/HeJ mice (approximately 90% of the microbial population) compared to the C3Bir IL-10^-/-^ mice. Proteobacteria and Bacteroidetes were found at relatively high proportional levels in the IL-10^-/-^ mice compared to WT mice throughout the study (**Supplementary Figures 1A, B**). Changes in abundance at the phylum level were minor over the 19 weeks of the study. In WT mice, Proteobacteria increased between weeks 4 and 7 (1% to 3%, p=0.05) and then decreased between weeks 12 and 19 (3% to 0.8%, p=0.03) (**Supplementary Figure 1B**). At the phylum level, there were no significant changes in the microbial composition of the IL-10^-/-^ mice throughout the 19 weeks of the study.

To better understand the dynamics of the microbiota, the relative abundance of the most prevalent genera was tracked between weeks 4 and 19 (**Supplementary Figure 2A**). With the exception of *Lactobacillus* and *Prevotella*, the remaining taxa in the WT mice showed significant increases (p ≤ 0.05) in abundance as the study progressed (**Supplementary Figures 2B, C**). By 19 weeks of age, members of the genus *Eubacterium* had expanded to represent 7.74% of the total population while the remaining genera remained collectively less than 10% of the total microbial community (**Supplementary Figure 2C**).

Consistent with the Shannon diversity index, there was no increase observed in the complexity of the cecal microbiota of the IL-10^-/-^ mice. In addition, multiple taxa (e.g., *Blautia, Burkholderia*, *Butyrivibrio, Dorea, Lactococcus, Oscillibacter, Pseudomonas*, and *Roseburia)* detected at 4 weeks of age decreased in relative abundance throughout the duration of the study (**Supplementary Figures 2D, E**). Of these genera, *Lactococcus* and *Roseburia* are generally considered beneficial to mucosal health. In contrast, there was a significant increase in *Escherichia* from 2% to 8% of the total microbiota (p=0.004) between 12 and 19 weeks of age in the C3Bir IL-10^-/-^ mice. While the C3Bir IL-10^-/-^ mice were positive for *H. hepaticus* and *H. muridarum*, there was no evidence that there was an increase in *Helicobacter* species over the 19 weeks of the study.

### Functional Analysis of the Microbiomes of IL-10^-/-^ and WT Mice

**Figure 2:**
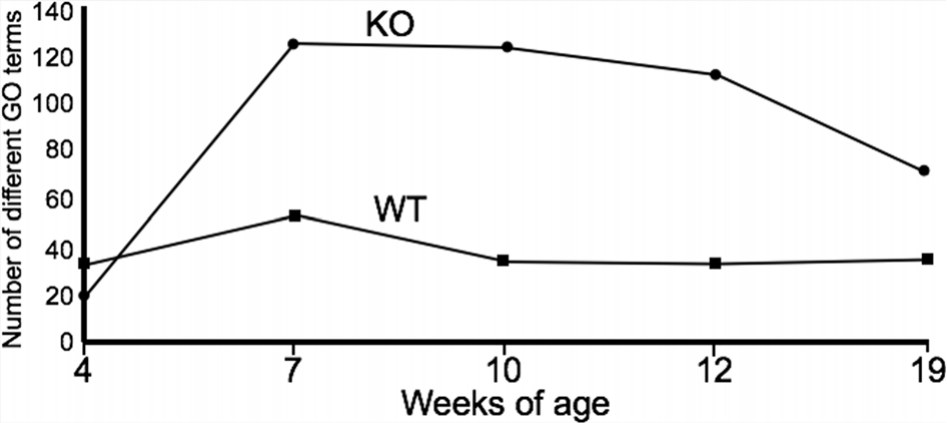
Summary of PICRUSt analysis. The number of GO terms are shown at each point of the study for WT and IL-10^-/-^ mice, as indicated. The number of mice used for each strain at each time point varied as follows: at 4 weeks of age, n = 5; at 7 weeks of age, n = 10; at 10 weeks of age, n = 9; at 12 weeks of age, n = 13; at 19 weeks of age, n = 5.

PICRUSt analysis was used to assess the extent to which differences in microbiota composition influenced predicted microbial functions over time. At week 4, the microbiota of the WT C3H/HeJ mice showed only a modest increase in the number of functional gene categories (GO categories) compared to the microbiota from the IL-10^-/-^ mice (**Figure 2**); however, there were fewer GO categories with LEfSe scores > 2 for the WT mice (**Supplementary Table 1**). For the duration of the study, the major functional changes in the WT microbiota remained relatively constant with only a few functional categories with LEfSe scores > 2 (**Supplementary Table 1A**). With respect to the microbiota of the C3Bir IL-10^-/-^ mice, an increase in the number of GO term categories was observed after week 7 weeks of age and these differences between WT and KO mice were largely maintained throughout the duration of the study (**Figure 2**). The functional categories whose representation showed marked changes in the IL-10^-/-^ mice, but not in WT mice, included metabolism, xenobiotic degradation, signal processing, biosynthesis of secondary metabolites, and DNA repair (**Supplementary Table 1B**). In the WT mice, the functional changes encompassed primarily carbohydrate and amino acid metabolism.

### Macroscopic/Microscopic Cecal Lesions Provide Evidence of Early Onset of Intestinal Inflammation

**Figure 3:**
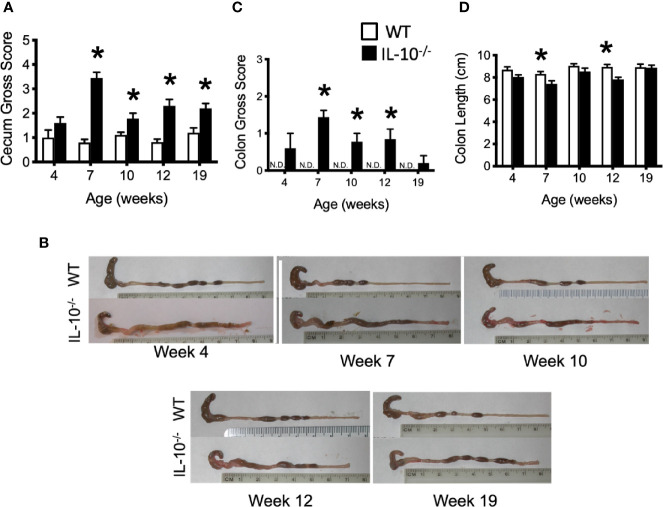
Macroscopic evaluation of GI tissue from WT and IL-10^-/-^ mice. Macroscopic lesion scores from WT (white bars) and IL-10^-/-^ (black bars) mice for the cecum **(A)** and colon **(C)**. Representative photomicrograph depicting colons from WT and IL-10^-/-^ mice at each time point as indicated **(B)**. **(D)** Graphical comparison of colon lengths. Data are presented as mean ± SEM. * = p ≤ 0.05 comparing WT and IL-10^-/-^ for each time point. The number of mice used for each strain at each time point varied as follows: at 4 weeks of age, n = 5; at 7 weeks of age, n = 10; at 10 weeks of age, n = 9; at 12 weeks of age, n = 13; at 19 weeks of age, n = 5.

To connect the observed differences in the microbiota between the IL-10^-/-^ and C3H/HeJ mice with changes in mucosal health, we compared differences in the expression of host inflammatory responses between the C3H/HeJ mice and the C3Bir IL-10^-/-^ mice over the course of 19 weeks. While no significant differences were observed in macroscopic cecal lesions between the C3H/HeJ and IL-10^-/-^ mice at 4 weeks of age, the IL-10^-/-^ mice had significantly (p ≤ 0.05) greater macroscopic lesion scores (range 0-5) at all subsequent time points (**Figure 3A**). The observed typhlocolitis of the IL-10^-/-^ mice was characterized by the presence of intraluminal blood, tissue thickening, cecal atrophy, and enlarged lymphoid aggregates (**Figure 3B**). Similarly, the colons from the IL-10^-/-^ mice presented with higher macroscopic lesion scores (range 0–3) at weeks 7, 10, and 12 compared to C3H/HeJ mice (**Figure 3C**) that were characterized by colonic tissue thickening and diarrheic luminal contents. Colon lengths of IL-10^-/-^ mice were found to be significantly (p ≤ 0.05) shorter at weeks 7 and 12 (**Figure 3D**). In contrast, colons from C3H/HeJ mice displayed no cecal or colonic macroscopic lesions at any point in the study.

**Figure 4:**
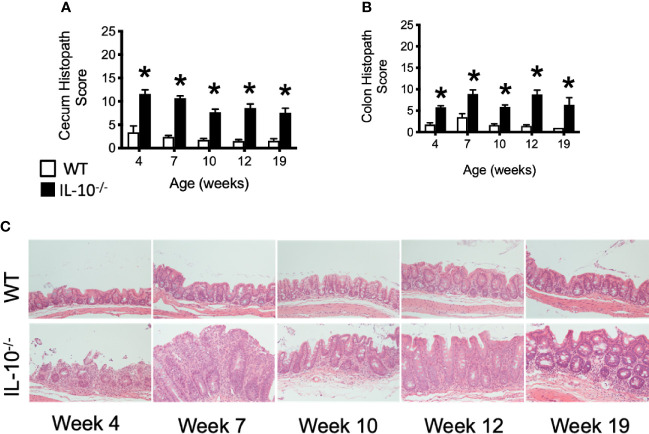
Microscopic evaluation of GI tissue from WT and IL-10^-/-^ mice. Microscopic lesion scores for mice for the cecum **(A)** and colon **(B)**. Data are presented as mean ± standard error of the mean (SEM) and for statistical comparison, * = p ≤ 0.05 when comparing WT and IL-10^-/-^ mice at a given time point. See *Materials and Methods* for the criteria used to generate the lesion scores. **(C)** Representative photomicrographs of cecal tissue collected from WT and IL-10^-/-^ mice at different time points. Tissue samples were fixed in 10% buffered formalin and routinely processed prior to staining with hematoxylin and eosin. All images were magnified 200X. The number of mice used for each strain at each time point varied as follows: at 4 weeks of age, n = 5; at 7 weeks of age, n = 10; at 10 weeks of age, n = 9; at 12 weeks of age, n = 13; at 19 weeks of age, n = 5.

Microscopic examination of both cecal and colonic tissues revealed statistically significant (p ≤ 0.05) levels of inflammation present in the IL-10^-/-^ mice at all time points compared to the tissue from C3H/HeJ mice (**Figures 4A, B**). As shown in **Figure 4C**, cecal tissue sections from IL-10^-/-^ mice had noticeable mucosal hyperplasia, mononuclear cell infiltrates in the lamina propria, and a loss of goblet cells, that was not observed in the cecal tissue of C3H/HeJ mice.

### Serum Pro-Inflammatory/Chemotactic Cytokines and SAA Are Increased in IL-10^-/-^ Mice Versus WT Mice

**Figure 5:**
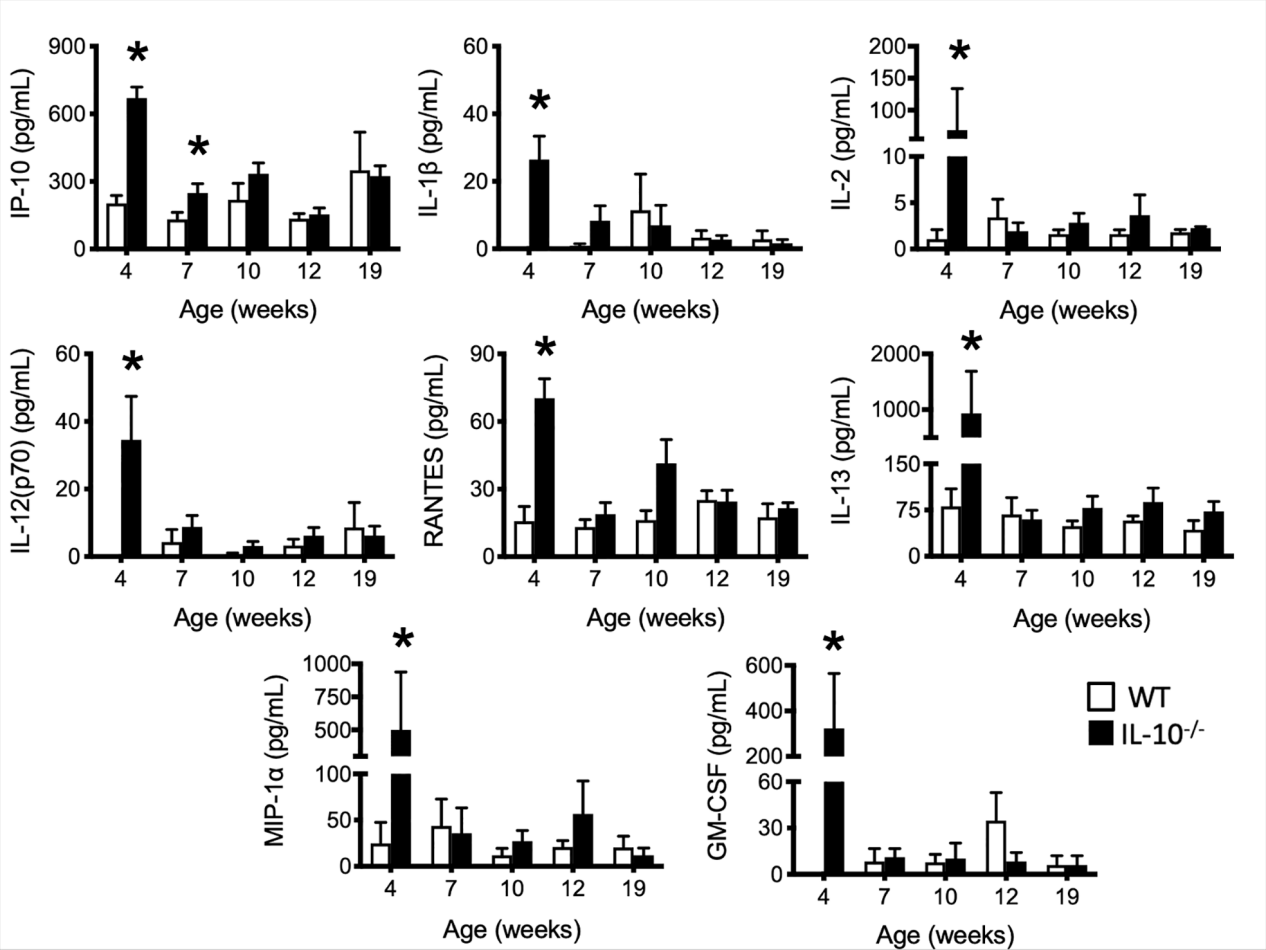
Levels of selected serum cytokines and chemokines elevated at early time points in the serum of IL-10^-/-^ mice. Immune factors in serum samples collected between four and 19 weeks of age that were assayed included: IP-10, IL-1β, IL-2, IL-12(p70), RANTES, IL-13, MIP-1α and GM-CSF. Serum samples were collected from WT (white bars) and IL-10^-/-^ (black bars) mice. Data are presented as mean ± SEM. *= p ≤ 0.05 comparing samples between WT and IL-10^-/-^ mice at each time point. The number of mice used for each strain at each time point varied as follows: at 4 weeks of age, n = 5; at 7 weeks of age, n = 10; at 10 weeks of age, n = 9; at 12 weeks of age, n = 13; at 19 weeks of age, n = 5.

**Figure 6:**
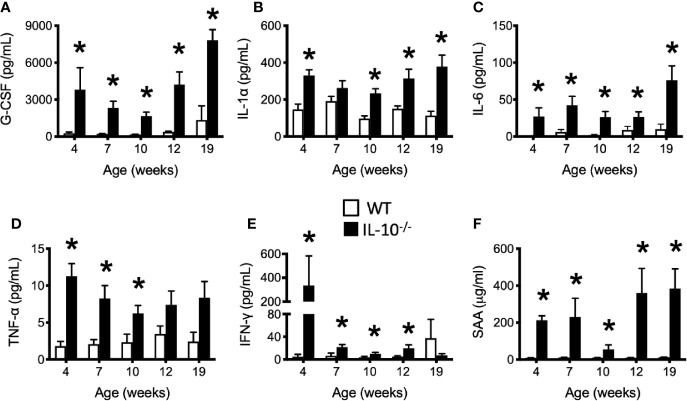
Levels of serum cytokines and chemokines that were persistently elevated in the serum of IL-10^-/-^ mice. Immune factors assayed in serum samples collected between 4 and 19 weeks of age include: **(A)** G-CSF; **(B)** IL-1α; **(C)** IL-6; **(D)** TNF-α **(E)** IFN-γ; and **(F)** serum amyloid A (SAA). Serum samples were collected from the IL-10^-/-^ mice (black bars) and WT mice (white bars). Data are presented as mean ± SEM. * = p ≤ 0.05 comparing WT and IL-10^-/-^ for each individual time point. The number of mice used for each strain at each time point varied as follows: at 4 weeks of age, n = 5; at 7 weeks of age, n = 10; at 10 weeks of age, n = 9; at 12 weeks of age, n = 13; at 19 weeks of age, n = 5.

**Figure 7:**
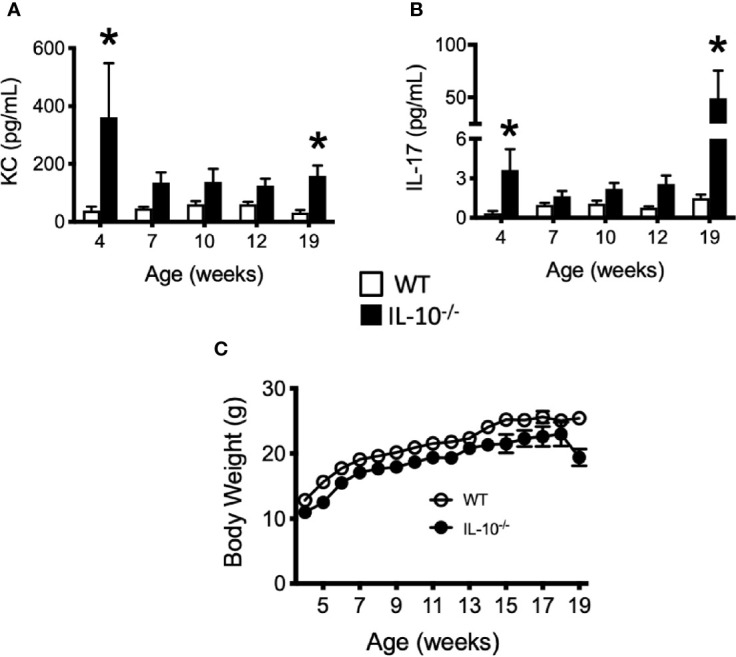
Levels of cytokine and chemokines that were elevated in the serum of IL-10^-/-^ mice at 19 weeks of age. Concentrations of KC **(A)** and IL-17 **(B)** were measured in the serum of wild type (white bars) an IL-10-/- mice. Whole body weights of WT and IL-10^-/-^ mice during the 19 week trial **(C)**. Data are presented as mean ± SEM. Both groups of mice gained a similar amount of weight between weeks 4 and 18 of age (IL-10^-/-^ = 12.0 g, WT = 12.2 g). At each time point, there were equal numbers of WT and IL-10^-/-^ mice included in the analysis. The number of mice used for each strain at each time point varied as follows: at 4 weeks of age, n = 5; at 7 weeks of age, n = 10; at 10 weeks of age, n = 9; at 12 weeks of age, n = 13; at 19 weeks of age, n = 5.

To investigate the progression of the systemic inflammatory response of IL-10^-/-^ mice over time, concentrations of pro-inflammatory cytokines and chemokines were measured in serum samples as biomarkers of inflammation. At 4 weeks of age, there were already significant elevation of chemokines and cytokines (e.g., IP-10, IL-1β, IL-2, IL-12(p70), RANTES, IL-13, MIP-1α, and GM-CSF) in the serum of the IL-10^-/-^ mice but these elevated levels did not persist over the course of the experiment (**Figure 5**). Over the course of the 19 weeks, significantly (p ≤ 0.05) elevated concentrations of G-CSF, IL-1α, IL-6, and TNF-α (**Figures 6A–D**) were observed in the serum of the IL-10^-/-^ mice in comparison to C3H/HeJ mice. IFN-γ levels were significantly (p ≤ 0.05) elevated in IL-10^-/-^ mice at each time point except week 19 (**Figure 6E**). As an acute phase protein observed during inflammation, SAA was significantly increased (p ≤ 0.05) in the serum of IL-10^-/-^ mice compared to C3H/HeJ mice at all-time points (**Figure 6F**). KC and IL-17 were the only two cytokines/chemokines that were elevated at week 4 (during the initial inflammatory surge) and then again at week 19 (**Figures 7A, B**) when severe weight became apparent in the IL-10^-/-^ mice and the experiment was terminated (**Figure 7C**).

## Discussion

Throughout the experiment, the C3Bir IL-10^-/-^ mice presented with severe, histopathological typhlocolitis. This is consistent with previous studies using this IL-10^-/-^ mouse model (3, 4). Unexpectedly, cecal inflammation was already present at 4 weeks of age and was associated with the increased expression of serum cytokines/chemokines (IP-10, IL-1β, IL-2, IL-12(p70), RANTES, IL-13, MIP-1α, GM-CSF) in the IL-10^-/-^ mice. There were also a subset of cytokines/chemokines that were chronically elevated throughout the duration of the experiment: G-CSF, IL-1α, IL-6, TNF-α, and IFN-γ. These observations in the presence of serum cytokines/chemokines emphasizes the dynamic nature of chronic inflammation and suggests that these different cytokines may contribute temporally to the initiation, maintenance, and/or attempts to resolve chronic inflammation in IL-10^-/-^ mice (57). KC and IL-17 interestingly were elevated at both the onset of disease (4 weeks) and when the mice developed clinical disease at 19 weeks of age; however, these cytokines were not significant elevated at other timepoints. The observed profile of cytokine production dovetail nicely with reports from others regarding elevated cytokine levels in IL-10^-/-^ mice. A study by Buchler et al. which also used IL-10^-/-^ mice on a C3Bir background also showed increased levels of IL-6, IL-17, and IFN-γ (4). Work by Matharu et al. also implicate the secretion of both IL-17 and IFN-γ from dysregulated CD4^+^ regulatory T cells (T_REG_) in their model of IL-10^-/-^ colitis where the mice were deficient in both IL-10 and TLR4 (58). These results demonstrate the importance of an effective T_REG_ cell response in maintaining mucosal health and highlights the cytoprotective benefits of TLR signaling. IL-10 and IL-17 were also implicated in the disease process by Eun et al. In their study, using germfree mice inoculated with seven bacterial species from an IBD patient, they examined the degree of inflammation in B6 and 129 IL-10^-/-^ mice at two separate time points (59). As previously mentioned, this study also revealed that the severity of inflammation was greater in the 129 IL-10^-/-^ mice which correlated with the induction of antigen-specific IFNγ and IL-17 at six and 12 weeks post-colonization.

The severity of inflammation that develops in IL-10^-/-^ mice is dependent on the composition of the intestinal resident microbiota (60). Using a model of *Helicobacter-*induced IL-10^-/-^ mouse of colitis, Yang and colleagues demonstrated that mice housed within different facilities developed colitis with characteristically different levels of inflammation. Using a 129/SvEv IL-10^-/-^ mouse model of colorectal cancer, there is evidence that the nature of intestinal inflammation elicits changes in the microbiota (61). Based on the analysis of beta-diversity in the microbiota, we demonstrated that the dysbiosis observed in the IL-10^-/-^ mice was associated with a failure to establish a diverse microbiota as was observed in the C3H/HeJ mice. The microbiota of both C3H/HeJ and IL-10^-/-^ mice was of comparable complexity at four weeks of age, which may not have been expected given the results obtained at later time points. However, after four weeks of age, the microbiota of the C3H/HeJ mice increased in diversity to levels never attained by the IL-10^-/-^ mice. As the C3H/HeJ mice aged, bacteria generally recognized as beneficial, such *Eubacterium, Lactococcus, and Ruminococcus*, significantly (p ≤ 0.05) increased in abundance and in Shannon diversity. This was not observed in the IL-10^-/-^ mice and this lack of diversity and richness is similar to that observed by Maharshak and colleagues at four weeks following colonization of germfree IL10^-/-^ mice with a SPF microbiota (39, 42).

At four weeks of age, the presence of elevated levels of pro-inflammatory cytokines was observed in the serum of the IL-10^-/-^ mice. These observations suggest that there is a critical time where inflammation appears to interfere with the natural development or diversification of the microbiota. To our knowledge, this has not been previously reported. Despite the general appearance of health between four and 17 weeks of age, the C3Bir IL-10^-/-^ mice maintained a dysbiotic profile of their microbiota throughout the study; in other words, the dysbiosis did not abruptly develop with the onset of marked weight loss at 19 weeks of age. Consequently, it is likely that the reduced abundance of key taxa contributed to the chronicity and severity of colitis. For example, taxonomic characterization of the microbiota revealed that the IL-10^-/-^ mice experienced a general decrease in abundance of bacteria regarded as beneficial (e.g., *Blautia*, *Lactococcus*, and *Roseburia*) while the abundance of Proteobacteria (e.g., *E. coli*) increased. Similar increases in Proteobacteria have been noted in other IL-10^-/-^ studies as well (39, 42). While the C3Bir IL-10^-/-^ mice were colonized by *H. hepaticus* and *H. muridarum,* there was no demonstrable increase in *Helicobacter* species over the 19 weeks of this study.

The development of the composition of the microbiota over time was hugely distinct between inflamed vs non-inflamed gastrointestinal tracts. The rarefaction analysis of observed species and the Shannon diversity index revealed an ordered increase in microbial diversity as the C3H/HeJ mice aged. In contrast, the C3Bir IL-10^-/-^ mice showed only a limited increase in the diversity of the observed microbial species over time. The progression of microbial diversity may have been significantly restricted as a consequence of the elevated levels of pro-inflammatory cytokine/chemokines seen at four weeks of age. As mentioned above, similar results were obtained in the study by Maharshak et al. which colonized germfree WT and IL-10^-/-^ mice with a SPF microbiota (39). Using fecal pellets as their source material, they reported a decrease in both microbial richness and diversity over time in the IL-10^-/-^ mice. In their WT mice, they reported increased bacterial richness over time supporting the data in this paper. In contrast, when Redhu et al. compared the fecal microbiota in IL-10 receptor knockout mice, they observed no differences in microbial diversity between the WT and IL-10R^-/-^ mice over time (41).

Identification of specific taxa from 16S rRNA gene sequences also revealed substantial differences in the richness of the microbial composition at the phylum and genus levels. Statistically significant (p ≤ 0.05) decreases in several genera in the C3Bir IL-10^-/-^ mice were observed over the course of the study. At 4 weeks of age, *Blautia, Butyrivibrio, Lactococcus, Roseburia*, and *Ruminococcus* spp. comprised 46% of the microbial community in the C3Bir IL-10^-/-^ mice but, by 7 weeks of age, these species represented only 21% of the microbiota support the impact of chronic inflammation on the composition of the intestinal microbiota. Furthermore, these taxa include members of the *Lachnospiraceae* family that is part of the *Clostridium coccoides* cluster (cluster XIVa) group which have been shown to be beneficial to intestinal health (7, 62). Reduced microbial diversity has been well documented both in human and companion animal IBD patients (20, 42, 63). For example, studies have shown that reduction of species belonging to the *C. coccoides* cluster is a common feature in the microbial communities colonizing the colon of human IBD patients (7, 62, 64–67). In a study evaluating IBD in twins, *Roseburia* spp. were decreased in both the feces and ileal biopsy samples of patients with ileal CD compared to healthy controls (13).

In addition to reductions of specific members of the community, there are increases in certain microbes associated with IBD as well. In particular, increased abundance of *Enterobacteriaceae* has been linked to the pathogenesis of mucosal inflammation in diverse species including humans, rodents, dogs, and cats (17, 63, 68–70). As has been noted in IBD patients, a significant increase in the abundance of *Enterobacteriaceae* was observed in the IL-10^-/-^ mice at 19 weeks of age and as the mice began to develop clinical signs of disease (i.e., weight loss) which was also accompanied by increased levels of IL-17 and KC in their serum. In these current studies, the C3Bir IL-10^-/-^ mice abruptly lost greater than 10% of their body weight and were removed from the study for humane reasons (**Figure S3**). IL-17 is increased in humans with IBD and has been associated with intestinal inflammation in several animal models (42, 71, 72). Previous studies using the IL-10^-/-^ model of colitis have either used denaturing gradient gel electrophoresis (DGGE) for their analysis of microbial compositional (40, 42) or have only collected fecal pellets for 4 weeks post-colonization of germ-free mice with a SPF microbiota or at 8 and 10 weeks of age (39). In contrast to the present work, these previous studies provide limited information regarding the dynamic changes of the microbial diversity or the inflammatory responses over time.

Using PICRUSt analysis, these results revealed functional differences in the microbiota between groups of mice that corresponded with the difference in environmental conditions encountered by the cecal microbial communities. C3H/HeJ mice exhibited fewer marked changes in functional categories over time compared to age matched IL-10^-/-^ mice suggesting that a more orderly maturation of the gut microbiota had occurred in the WT mice. Inspection of functional categories with LEfSe scores > 2 revealed a number of gene functions in IL-10^-/-^ mice that were not significantly altered in C3H/HeJ mice including DNA recombination, replication and repair mechanisms (week 7), signal transduction, cell motility and chemotaxis (week 12), and biosynthesis of secondary metabolites and xenobiotics degradation and metabolism (week 19). These differences likely represent adaptations in the functions of the microbial community in the C3Bir IL-10^-/-^ mice in response to the chronic gastrointestinal inflammation and the use of metabolic biomarkers may represent useful biomarkers to better understand the role of inflammation in shaping the gut microbiota.

Based on the results of this study, we find that there is a window of opportunity for microbial progression that is crucial for the development of a diverse microbiome. Similar studies have shown this effect in infants given antibiotics (73). A Finnish study by Korpela et al. revealed that children prescribed macrolide antibiotics between 2-7 years of age had a reduction in microbial richness even 24 months after antibiotic exposure (74). A longitudinal study by Yassour et al. that utilized metagenomics found that children given antibiotics had less diverse and less stable microbial communities compared to untreated children (75). These studies support our conclusion that if the progression of the microbiota is hindered (through inflammation or antibiotic usage) the effects are long-term with regards to the stability and diversity of the microbiome.

In conclusion, this study tracked both inflammation and cecal microbial composition temporally in C3Bir IL-10^-/-^ mice over multiple time points using 16S rRNA gene amplicon sequencing. Results indicate that the C3Bir IL-10^-/-^ mice had begun to develop intestinal inflammation as early as 4 weeks of age when their microbial diversity was at its greatest. The results obtained in this study suggests that intervention treatments, such as administration of prebiotics and/or probiotics, or anti-inflammatory therapies, may be more effective when administered at a critical time point to establish or maintain a beneficial microbiota contributing to improved gut health and clinical outcomes.

## Data Availability Statement

The raw data underlying the figures and tables associated with this manuscript have been made publically available. The short read sequences related to the analysis of the microbiota is deposited with NCBI under the BioProjects tab with an accession number of PRJNA231086. The ordinal data for the graphical data is deposited with Open Science Framework (doi:10.17605/OSF.IO/QT8GH).

## Ethics Statement

Before any animal related work was performed, the animal studies were reviewed and approved by Institutional Animal Care and Use Committee, Iowa State University, Ames, Iowa, USA.

## Author Contributions

A-MO performed the animal studies, collected data, and generated the initial figures and first draft. AR-T, AJ, and GP were involved in experimental design, data interpretation, and manuscript editing. CW performed statistical analysis. JS analyzed genomic data, generated figures and KO term analyses. JH performed the blinded histopathological analyses and contributed to data interpretation and discussion. MW was supervisor in charge of project over-sight, data analysis, writing and editing, and financial support of the project along with AJ and GP. All authors contributed to the article and approved the submitted version.

## Funding

This work was supported in part by internal research grants provided by Iowa State University, College of Veterinary Medicine and Graduate College as well as funds provided by the National Institute of General Medicine (R01GM099537.

## Conflict of Interest

The authors declare that the research was conducted in the absence of any commercial or financial relationships that could be construed as a potential conflict of interest.

## Supplementary Material

Supplementary Figure 1 Taxonomic composition of the cecal microbiota at the phylum level. **(A)** Phylum level composition of WT and IL-10^-/-^ mice, as indicated, throughout the study. **(B)** Specific phyla with statistically significant changes in abundance over the course of the study from WT and IL-10^-/-^ mice. The relative abundance (%) for each group is shown and p-values are shown parenthetically. The number of mice used for each strain at each time point varied as follows: at 4 weeks of age, n = 5; at 7 weeks of age, n = 10; at 10 weeks of age, n = 9; at 12 weeks of age, n = 13; at 19 weeks of age, n = 5.

Supplementary Figure 2 Taxonomic composition of the cecal microbiota at the genus level. **(A)** Genus level composition of WT and IL-10^-/-^ mice cecal microbiome throughout the study. **(B** and **D)**. The % abundance of specific bacterial genera from WT and IL-10^-/-^ mice are shown by the colored lines and identified by the key. **(C** and **E)**. Tables list specific genera with statistically significant changes in abundance over the course of the study from WT and IL-10^-/-^ mice. The relative abundance (%) for each group is shown and p-values are shown parenthetically. The number of mice used for each strain at each time point varied as follows: at 4 weeks of age, n = 5; at 7 weeks of age, n = 10; at 10 weeks of age, n = 9; at 12 weeks of age, n = 13; at 19 weeks of age, n = 5.

Supplementary Table 1 **(A)** LefSE scores for differentially abundant gene families from PICRUSt analysis. Functional categories and LefSE scores for WTmice. The number of mice used for each strain at each time point varied as follows: at 4 weeks of age, n =5; at 7 weeks of age, n = 10; at 10 weeks of age, n = 9; at 12 weeks of age, n = 13; at 19 weeks of age, n = 5. **(B)** LefSE scores for differentially abundant gene families from PICRUSt analysis. Functional categories and LefSE scores for IL-10^-/-^ mice. The number of mice used for each strain at each time point varied as follows: at 4 weeks of age, n = 5; at 7 weeks of age, n = 10; at 10 weeks of age, n = 9; at 12 weeks of age, n = 13; at 19 weeks of age, n = 5.
