# Supplementary figures and images for: Temporal Dynamics of Chronic Inflammation on the Cecal Microbiota in IL-10-/- Mice
Source: Front Immunol. 2021 Feb 16;11:585431. doi: 10.3389/fimmu.2020.585431 (PMC7921487; doi:10.3389/fimmu.2020.585431)

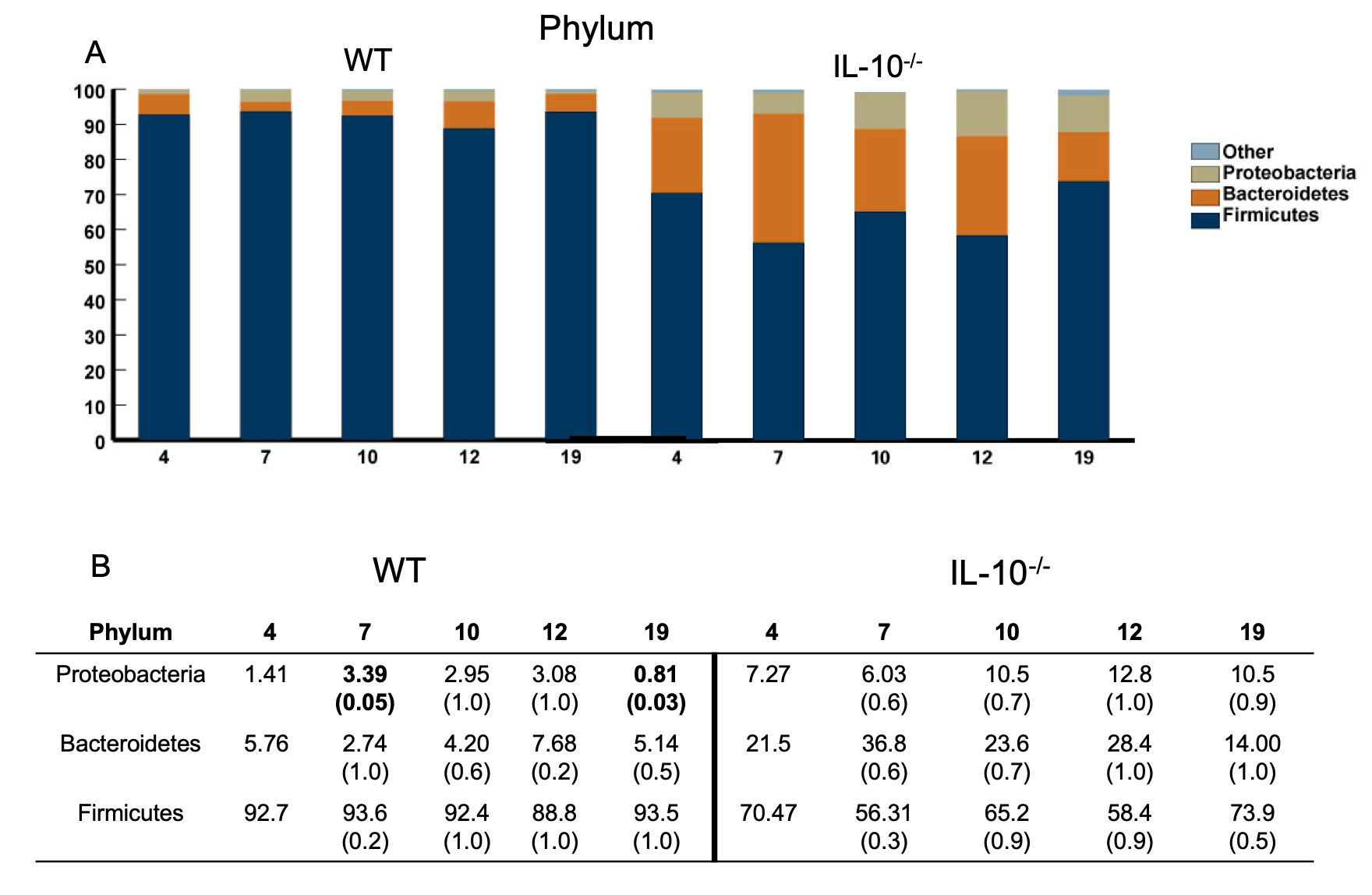

Supplement: Supplementary Figure 1 — Taxonomic composition of the cecal microbiota at the phylum level. (A) Phylum level composition of WT and IL-10-/- mice, as indicated, throughout the study. (B) Specific phyla with statistically significant changes in abundance over the course of the study from WT and IL-10-/- mice. The relative abundance (%) for each group is shown and p-values are shown parenthetically. The number of mice used for each strain at each time point varied as follows: at 4 weeks of age, n = 5; at 7 weeks of age, n = 10; at 10 weeks of age, n = 9; at 12 weeks of age, n = 13; at 19 weeks of age, n = 5. [file Image_1.tiff]

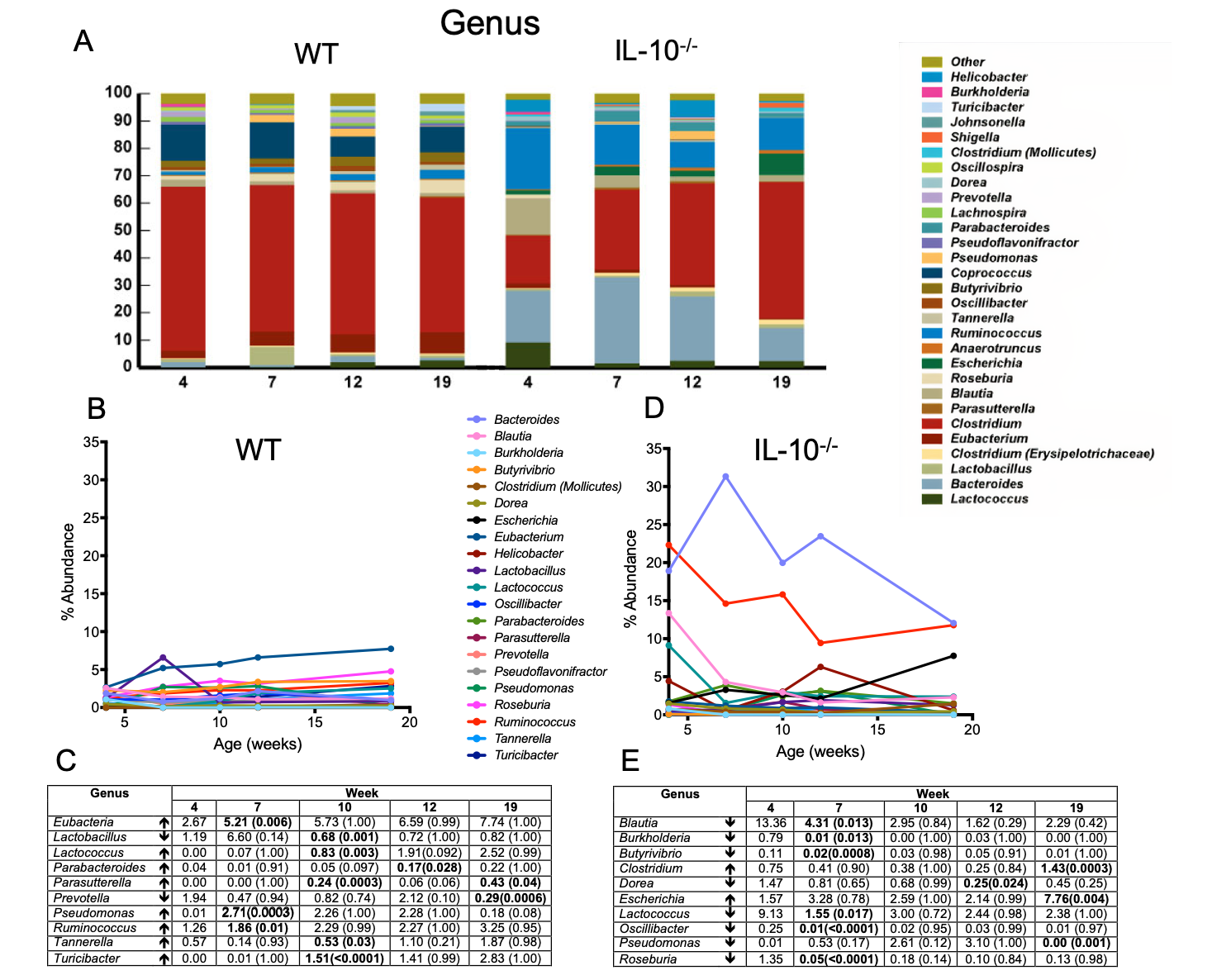

Supplement: Supplementary Figure 2 — Taxonomic composition of the cecal microbiota at the genus level. (A) Genus level composition of WT and IL-10-/- mice cecal microbiome throughout the study. (B and D). The % abundance of specific bacterial genera from WT and IL-10-/- mice are shown by the colored lines and identified by the key. (C and E). Tables list specific genera with statistically significant changes in abundance over the course of the study from WT and IL-10-/- mice. The relative abundance (%) for each group is shown and p-values are shown parenthetically. The number of mice used for each strain at each time point varied as follows: at 4 weeks of age, n = 5; at 7 weeks of age, n = 10; at 10 weeks of age, n = 9; at 12 weeks of age, n = 13; at 19 weeks of age, n = 5. [file Image_2.tiff]

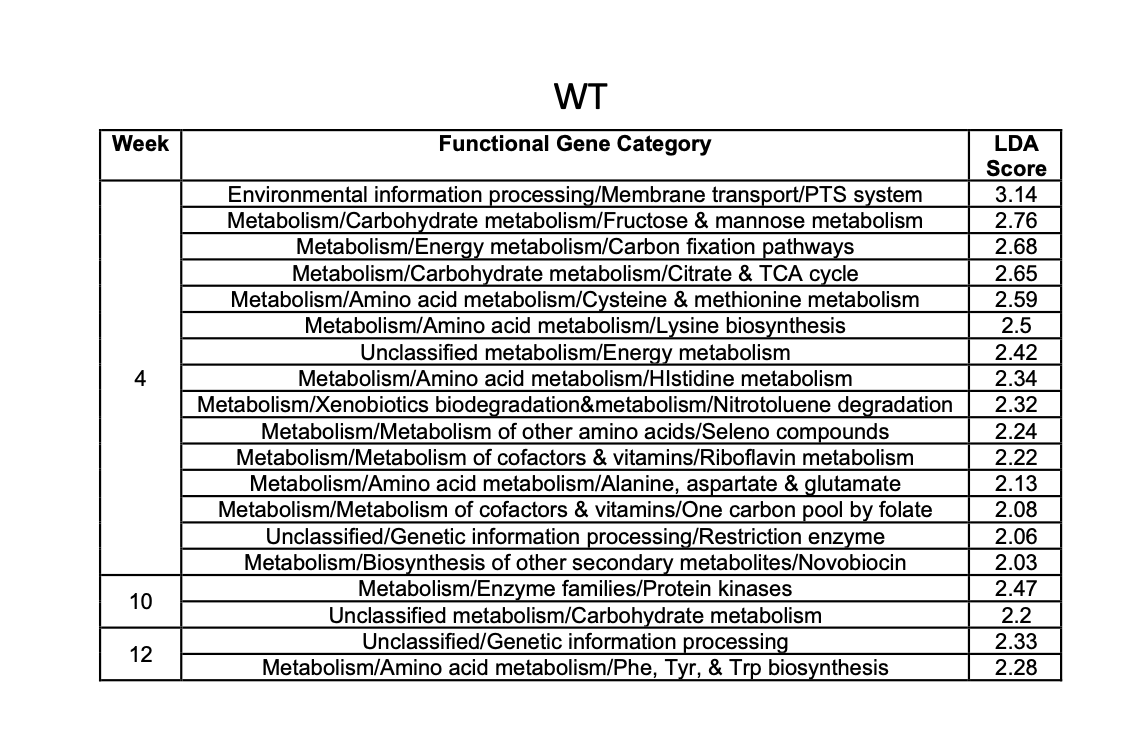

Supplement: Supplementary Table 1 — (A) LefSE scores for differentially abundant gene families from PICRUSt analysis. Functional categories and LefSE scores for WTmice. The number of mice used for each strain at each time point varied as follows: at 4 weeks of age, n =5; at 7 weeks of age, n = 10; at 10 weeks of age, n = 9; at 12 weeks of age, n = 13; at 19 weeks of age, n = 5. (B) LefSE scores for differentially abundant gene families from PICRUSt analysis. Functional categories and LefSE scores for IL-10-/- mice. The number of mice used for each strain at each time point varied as follows: at 4 weeks of age, n = 5; at 7 weeks of age, n = 10; at 10 weeks of age, n = 9; at 12 weeks of age, n = 13; at 19 weeks of age, n = 5. [file DataSheet_1.zip › Supplementary Table 1a.TIFF]

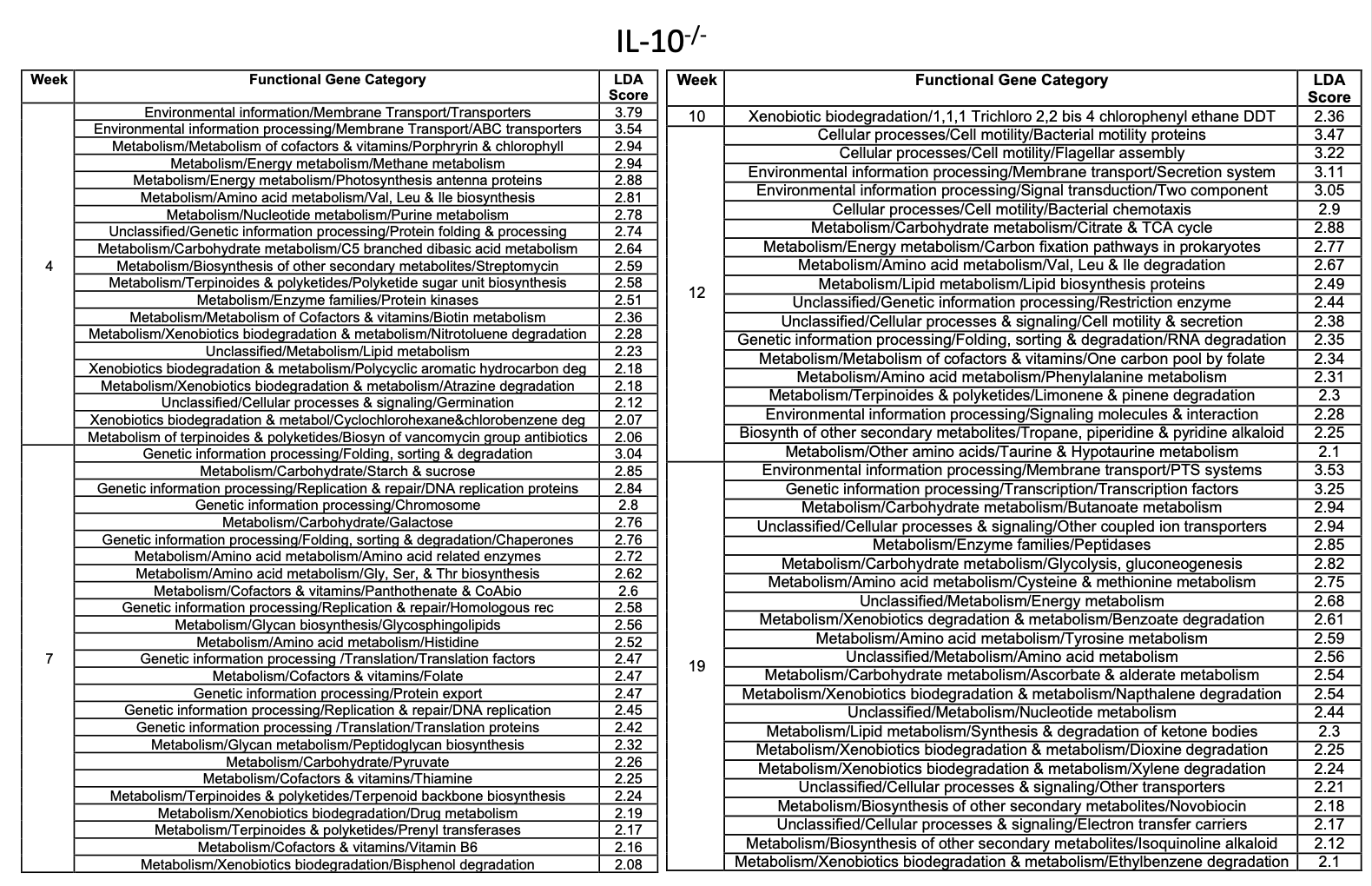

Supplement: Supplementary Table 1 — (A) LefSE scores for differentially abundant gene families from PICRUSt analysis. Functional categories and LefSE scores for WTmice. The number of mice used for each strain at each time point varied as follows: at 4 weeks of age, n =5; at 7 weeks of age, n = 10; at 10 weeks of age, n = 9; at 12 weeks of age, n = 13; at 19 weeks of age, n = 5. (B) LefSE scores for differentially abundant gene families from PICRUSt analysis. Functional categories and LefSE scores for IL-10-/- mice. The number of mice used for each strain at each time point varied as follows: at 4 weeks of age, n = 5; at 7 weeks of age, n = 10; at 10 weeks of age, n = 9; at 12 weeks of age, n = 13; at 19 weeks of age, n = 5. [file DataSheet_1.zip › Supplementary Table 1b.TIFF]
